# P-1070. Antimicrobial Susceptibility Trends and Mortality of Pseudomonas aeruginosa Hospital-acquired Pneumonia and Ventilator-associated Pneumonia among Adult Patients: A Retrospective Single-center Study

**DOI:** 10.1093/ofid/ofaf695.1265

**Published:** 2026-01-11

**Authors:** Lara Monica S Ng, Cybele Lara R Abad

**Affiliations:** Philippine General Hospital, Davao, Davao del Sur, Philippines; University of the Philippines - Philippine General Hospital, Manila, National Capital Region, Philippines

## Abstract

**Background:**

*Pseudomonas aeruginosa* is a leading cause of nosocomial infections. This study examined the antimicrobial susceptibility trends (AST), clinical profile and outcomes of *P. aeruginosa* hospital-acquired and ventilator-associated pneumonia (HAP/VAP).

**Figure 1:**
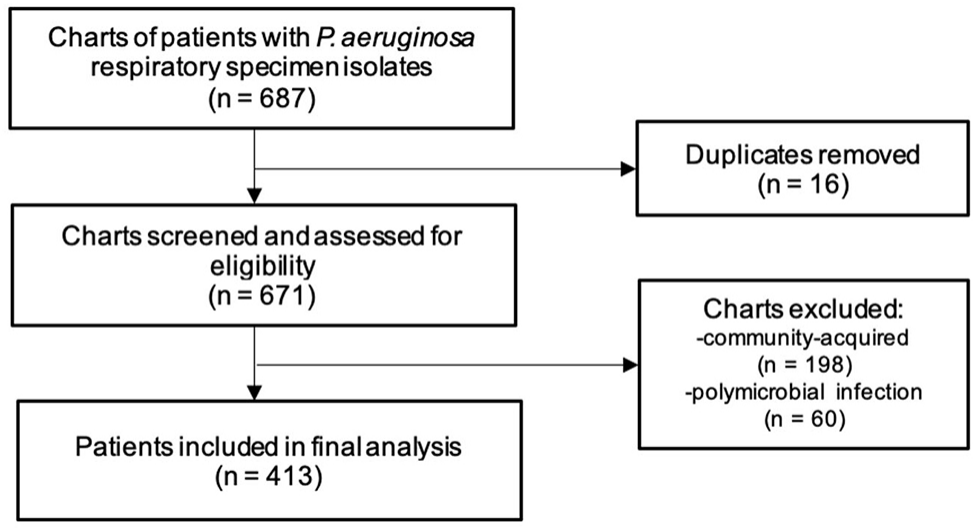
Flow diagram of study inclusion

**Table 1:**
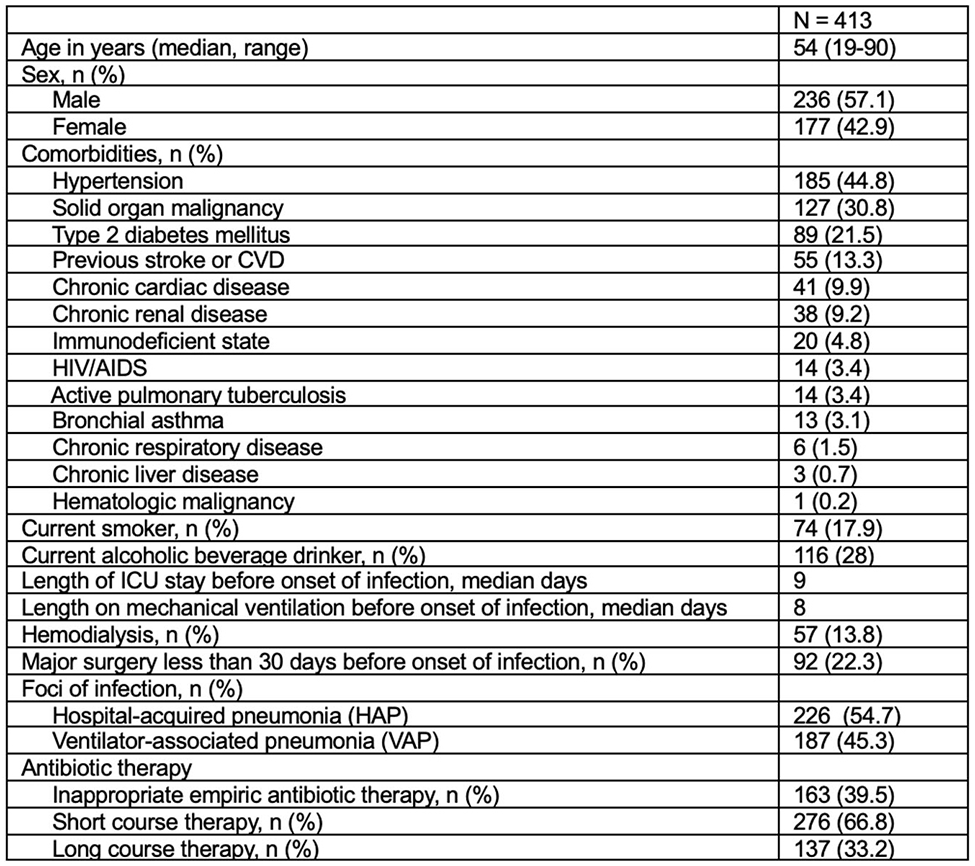
Demographic and clinical characteristics of adult patients with Pseudomonas aeruginosa HAP/VAP admitted to the Philippine General Hospital (2020-2024)

**Methods:**

A retrospective observational study of adults with *P. aeruginosa* HAP/VAP from 1/1/2020 to 8/31/2024 was undertaken. Relevant data were extracted from electronic records, and *P. aeruginosa* identified using VITEK 2 and MALDI-TOF technology. Continuous variables were expressed in median and range, and categorical variables as frequencies and percentages. The Mann–Whitney U test was used for non-parametric comparison of continuous variables, and the Chi-square test for univariate analysis of categorical variables. A binary logistic regression model identified factors associated with mortality in patients with *P. aeruginosa* HAP/VAP.

**Figure 2:**
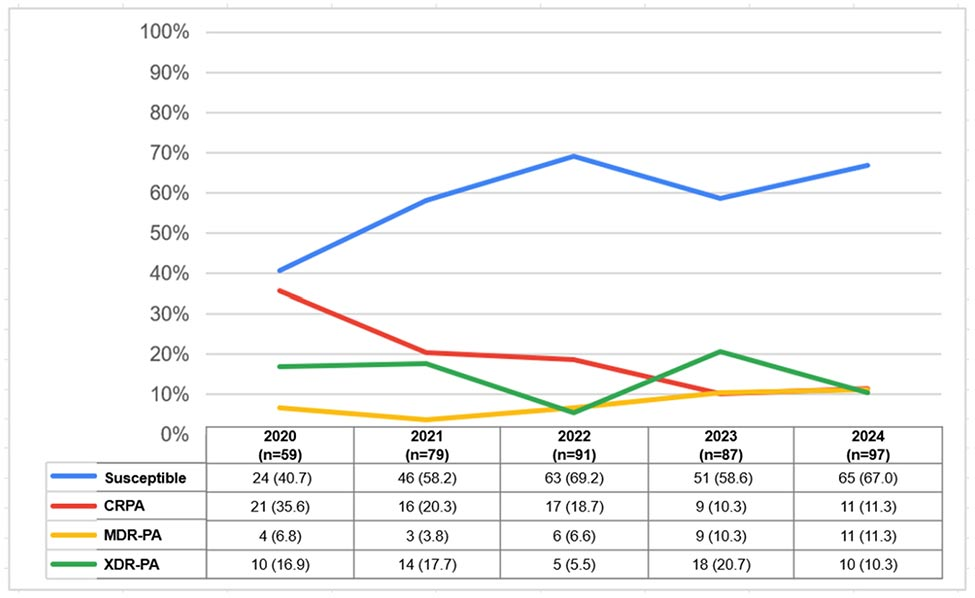
Proportion of susceptible and resistant Pseudomonas aeruginosa HAP/VAP isolates among adult patients admitted to the Philippine General Hospital over 5 years (2020-2024)

**Table 2:**
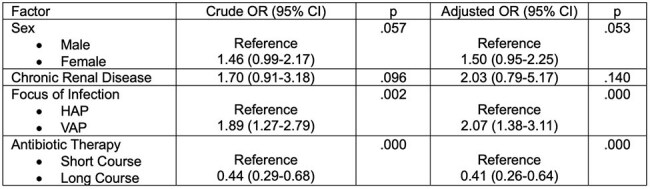
Univariate and Multivariate regression analysis on the factors associated with all-cause mortality among patients with Pseudomonas aeruginosa HAP/VAP

**Results:**

Of 687 patients, 413 were included in the final analysis (Fig. 1). The median age was 54 (range 19-90) years. The most common comorbidities were hypertension (185/413, 44.8%), solid organ malignancy (127/413, 30.8%), and type 2 diabetes mellitus (89/413, 21.5%) (Table 1). The proportion of patients with multi-drug resistant-PA (MDR-PA) increased, carbapenem-resistant (CRPA) decreased, and extremely drug-resistant (XDR-PA) fluctuated over time (Fig. 2). Although there was no significant change in AST, increasing susceptibility to carbapenems and decreasing susceptibility to piperacillin-tazobactam were observed. All-cause mortality rate was 46% (190/413), with 80% (152/190) attributed to *P. aeruginosa* HAP/VAP. A decreasing mortality trend was observed in patients with MDR-PA and XDR-PA HAP/VAP. Prolonged antibiotic therapy (≥14 days) was associated with better survival outcomes compared to short-course therapy (≤7 days) (p 0.000).

**Conclusion:**

In our study, *P. aeruginosa* HAP/VAP had high attributable mortality. However, a decreasing mortality trend was observed among patients with MDR-PA and XDR-PA HAP/VAP over the 5-year period, suggesting that factors other than antimicrobial resistance may influence mortality outcomes. Patients with *P. aeruginosa* HAP/VAP may benefit from prolonged antibiotic therapy.

**Disclosures:**

All Authors: No reported disclosures

